# Both *RAD5*-dependent and independent pathways are involved in DNA damage-associated sister chromatid exchange in budding yeast

**DOI:** 10.3934/genet.2017.2.84

**Published:** 2017-03-30

**Authors:** Michael T. Fasullo, Mingzeng Sun

**Affiliations:** College of Nanoscale Sciences and Engineering, SUNY Polytechnic Institute, 257 Fuller Road, Albany, New York 12203, United States

**Keywords:** DNA repair, DNA damage, template switching, budding yeast, homologous recombination

## Abstract

Sister chromatids are preferred substrates for recombinational repair after cells are exposed to DNA damage. While some agents directly cause double-strand breaks (DSBs), others form DNA base adducts which stall or impede the DNA replication fork. We asked which types of DNA damage can stimulate SCE in budding yeast mutants defective in template switch mechanisms and whether PCNA polyubiquitination functions are required for DNA damage-associated SCE after exposure to potent recombinagens. We measured spontaneous and DNA damage-associated unequal sister chromatid exchange (uSCE) in yeast strains containing two fragments of *his3* after exposure to MMS, 4-NQO, UV, X rays, and HO endonuclease-induced DSBs. We determined whether other genes in the pathway for template switching, including *UBC13*, *MMS2*, *SGS1*, and *SRS2* were required for DNA damage-associated SCE. *RAD5* was required for DNA damage-associated SCE after exposure to UV, MMS, and 4-NQO, but not for spontaneous, X-ray-associated, or HO endonuclease-induced SCE. While *UBC13*, *MMS2*, and *SGS1* were required for MMS and 4NQO-associated SCE, they were not required for UV-associated SCE. DNA damage-associated recombination between *his3* recombination substrates on non-homologous recombination was enhanced in *rad5* mutants. These results demonstrate that DNA damaging agents that cause DSBs stimulate SCE by *RAD5*-independent mechanisms, while several potent agents that generate bulky DNA adducts stimulate SCE by multiple *RAD5*-dependent mechanisms. We suggest that DSB-associated recombination that occurs in G2 is *RAD5*-independent.

## 1. Introduction

Sister chromatids are ideal templates for recombinational repair since they are essentially identical copies generated by DNA replication [1]. Higher levels of sister chromatid exchanges occur after cells are exposed to diverse DNA damaging agents [2] or in cell lines that are mutated for DNA metabolism genes, such as BLM, the gene mutated in Bloom's Syndrome [3]. Multiple genetic pathways, including those in recombinational repair [4], DNA damage-induced checkpoints [5]–[7], and nucleotide excision repair [8], are involved in promoting recombination between sister chromatids. While both recombinational repair and G2 checkpoint genes are required for double-strand break (DSB)-associated SCE [8],[9] and have been extensively studied (for review, see [10]), mechanisms that promote DNA damage-associated SCE after cells are exposed to DNA damaging agents that stall or impede DNA replication are still unclear.

Many potent recombinagens, which stimulate SCE, do not directly induce DSBs, but instead create DNA bulky adducts or intrastrand cross-links. Such agents include UV radiation [11], 4-nitroquinoline oxide (4NQO, [11]), and methyl methanesulfonate (MMS, [11],[12]). Specific types of bulky adducts can indirectly be a source of single-strand and DSBs by causing oxidative damage, by impeding DNA replication, or by being substrates for base excision (BER, [13]) or nucleotide excision repair enzymes (NER, [14],[15]). For example, MMS generates 3-methyl adenine adducts, which impedes DNA replication by blocking polymerases in the minor grove (for review, see [16]), and 4NQO generates stable quinoline monoadducts such as 3-(deoxyadenosin-N6-yl)-4AQO and N4-(guanosin-7-yl-4AQO) [17], which also stall DNA polymerase progression [18]. Whereas the 3-methyl adenine adducts are substrates for BER [19], 4-NQO-associated adducts are substrates for NER [14],[20]. However, additional studies support the notion that replication-associated SCE does not require NER [21], suggesting that bulky adducts may stimulate SCE by alternative mechanisms.

Such alternative mechanisms for stimulating SCE may involve DNA damage tolerance mechanisms, which allow DNA polymerases to bypass DNA adducts so that replication can be completed [22]–[24]. While polymerase-switch pathways, in which high fidelity polymerases are switched to low fidelity polymerases, are often error-prone, template switch mechanisms, in which the DNA polymerase switches from the damaged DNA template to the undamaged template, is error-free [25]–[27]. An intermediate in the template switch pathway is the Holliday structure, which can be resolved by Sgs1 (BLM) so that cross-over events are minimized [28],[29].

In eukaryotic cells, the *RAD18*/*RAD6* pathway confers DNA damage tolerance and catalyzes post-translational modifications in PCNA (for reviews, see [30],[31]). Rad6/Rad18 is required for monoubiquitination of PCNA, whereas Rad5, a ring finger protein that contains both a ATPase and an E3 ubiquitin domain, is required for subsequent PCNA polyubiquitination (for review, see [32]). The ATPase domain is required for Rad5's helicase activity, which is necessary to reverse collapsed replication forks so that template switch mechanisms can allow polymerases to proceed [33]. To add polyubiquitin to PCNA, the Rad5 E3 Ub ligase requires the Ubc13/Mms2 heterodimer, which functions as a E2 Ub-conjugating enzyme [30],[31]. Since neither *ubc13* nor *mms2* mutants are as UV sensitive as *rad5* mutants, other Rad5 functions, such as Rad5-helicase, are critical for UV resistance [34]. Nonetheless, the role of many *RAD5*-associated functions in DNA damage-associated or spontaneous SCE events is unclear.

While both *in vivo* and *in vitro* studies clearly support *RAD5*'s function in template switch mechanisms, it is unclear which types of DNA damage-associated SCE are actually mediated by *RAD5*. Previous experiments indicate that all DNA damage-associated unequal SCE (uSCE) require *RAD52*, *RAD55*, *RAD57* and *RAD51*, genes which are also required for DSB repair [4],[8]. Herein, we demonstrate that *RAD5* is required for DNA damage-associated SCE after exposure to potent recombinagens that do not directly cause DSBs, while *RAD5* is not required for DNA damage-associated uSCE after exposure to DSBs. While both *UBC13* and *MMS2* are required for MMS and 4NQO-associated uSCE, UV-associated uSCE is both *UBC13* and *MMS2*-independent. These studies indicate that there are multiple *RAD5*-dependent mechanisms for DNA damage-associated uSCE events, but that DSB-associated uSCE is *RAD5*-independent.

## 2. Materials and methods

### 2.1. Media and yeast strains

Standard media for the culture of yeast, SC (synthetic complete, dextrose), SC-HIS (SC lacking histidine), SC-LEU (SC lacking leucine), SC-TRP (SC lacking tryptophan), SC-URA (SC lacking uracil), YP (yeast extract, peptone), and YPD (YP, dextrose), are described by Burke et al. [35]. YPL medium contains YP with 2% lactate (pH 5.8); YPGlu medium contains YP medium with 2% glucose; YPGal medium contains YP medium with 2% ultra-pure galactose (Sigma, St Louis, MO).

### 2.2. Relevant yeast strains and recombination assays

**Figure 1.**
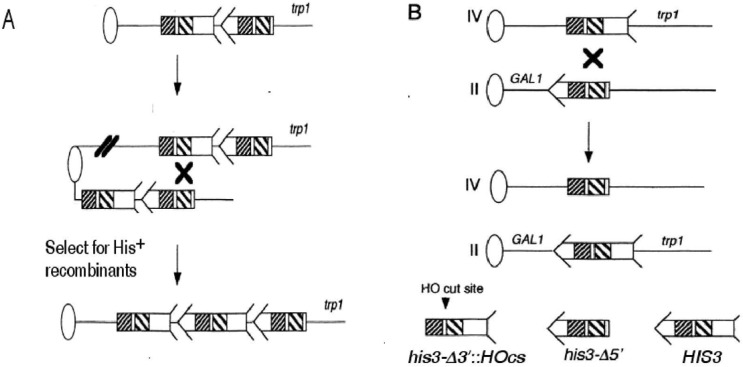
Recombination assays used in this study. Ovals represent centromeres and lines represent chromosomes. For simplicity, the left arms of the chromosomes are not included. The position and orientation of the *his3* recombinational substrates, which are present in strains used to measure (A) unequal SCE and (B) reciprocal translocations, are shown. An X designates potential sites of crossovers, and the resulting chromosomal rearrangement is presented. An arrow and feathers denote *HIS3*. As indicated on the bottom right of the figure, the 5′ deletion, *his3-Δ5*′, lacks the feathers and the 3′ deletion, *his3-Δ3*′, lacks the arrow. The two regions of sequence identity shared by the *his3* fragments are indicated by decorated boxes; broadly spaced diagonal lines indicate a region of 300 bp, and tightly spaced diagonal lines indicate a region of 167 bp. The 117-bp HO cut site (*HOcs*), as indicated by an arrow, is located between these sequences within the *his3-Δ3*′*::HOcs* fragment. In strains measuring SCE, the *his3*-truncated fragments are integrated into the *trp1* locus on chromosome IV. In strains measuring ectopic recombination, the *his3-Δ3*′ is located within the *TRP1* gene. The products of the recombination event (right) are two chromosomal translocations; in one translocation, *CEN2* is linked to the long arm of chromosome IV and in the other, *CEN4* is linked to the long arm of chromosome II.

The recombination assays are shown in Figure 1, and the strains are listed in Table 1. Haploid strains used to measure SCE contain two overlapping *his3* fragments [12], positioned in tandem at *trp1*, and were derived from YB163. The assay consists of two truncated fragments of the *his3* gene that placed in tandem so that their wild-type ends are in juxtaposition. The 117 *MAT***a** sequence containing the HO endonuclease recognition sequence was placed within one of the fragments, *his3-Δ3′::HOcs*, so that DSB-associated SCE could be directly measured, as previously described [5],[8]. The assay to measure translocation formation consists of two fragments of *his3* positioned on chromosomes IV and II, respectively [12].

The *rad5, sgs1, ubc13, srs2*, and *mms2* mutants were made by the appropriate genetic crosses; the original knock-out strains were derived from BY4741 [36] (Table 1). Strains used to determine the frequency of recombination events stimulated by HO endonuclease-induced DSBs contained the *MAT***a**-inc allele, so that the only DSB occurred at the recombination substrate. The *his3-Δ200* allele was confirmed by PCR, using oligonucleotides previously published [8]; the *rad5* disruption allele was confirmed by PCR using 5′AAATCAAAATGAAGTAAAACCCCTC3′ and 5′TGGC-TGGAAAACTTTCATCTACTAC3′, which flank the 5′ side and 3′ side of the gene, respectively.

The double *rad5 sgs1* and *rad5 srs2* mutants were made by genetic crosses and meiotic segregants were obtained after tetrad dissections. Two meiotic segregants were obtained that were *rad5 sgs1* and confirmed by PCR. The *rad5 sgs1* double mutant was also confirmed by hydroxyurea and UV sensitivity.

### 2.3. Measuring rates of spontaneous recombination

The rates (events per cell division) of spontaneous, mitotic events that generate either SCE or translocations were determined by the method of the median [37] as performed by Esposito et al. [38], using nine independent colonies for each rate calculation. At least two independent rate calculations were done for each strain, and statistical significance was determined by the Mann-Whitney U-test [39].

### 2.4. Determining frequencies of DNA damage-associated recombinants

Protocols used to measure the recombinogenicity of UV, X-rays, MMS, and 4-NQO were previously described [8],[11]. To measure radiation-associated recombination, we used an X-ray radiation source purchased from Faxitron, Inc. (Wheeling, IL), and the dose rate was 100 rad/min. A 254 nM germicidal lamp (2 J/M^2^/s) was used for UV irradiation. MMS and 4-NQO were purchased from Sigma-Aldrich Co.

**Table 1.** Yeast Strains.

Strain (Synonym)	Genotype	Autonomous plasmid	Reference (Source)
BY4741	*MAT***a** *ura3Δ0 leu2Δ0 his3Δ1 lys2Δ0 met15Δ0*		[36]
YA201	*See BY4741 rad5::KanMX*		[36]
YA284	*See BY4741 ubc13::KanMX*		[36]
YA285	*See BY4741 mms2::KanMX*		[36]
YA286	*See BY4741 sgs1::KanMX*		[36]
YA287	*See BY4741 srs2::KanMX*		[36]
YB163	*MAT**a**-inc ura3-52 his3*-*Δ200 ade2-101 lys2-801 trp1-Δ1 gal3 trp1::[his3-Δ3*′*::HOcs, his3*-*Δ5*′*]*		[8]
YB204	*MATα* *ura3-52 his3*-*Δ200 ade2-101 lys2-801 trp1-Δ1 gal3 trp1::[his3-Δ3*′*::HOcs, his3*-*Δ5*′*] leu2*-*Δ1*		[8]
YB441	*MATα* *ura3-52 his3*-*Δ200 ade2-101 trp1-Δ1 gal3 trp1::[his3-Δ3*′*::HOcs, his3*-*Δ5*′*] leu2*-*Δ1 rad5::KanMX*,		Meiotic segregant from YB204 × YA201
YB442	*MATα* *ura3-52 his3*-*Δ200 ade2-101 trp1-Δ1 gal3 trp1::[his3-Δ3*′*::HOcs, his3*-*Δ5*′*] leu2*-*Δ1 rad5::KanMX*,	PR-30	Leu^+^ transformant of YB441
YB443	*MATα* *ura3-52 his3*-*Δ200 ade2-101 trp1-Δ1 gal3 trp1::[his3-Δ3*′*::HOcs, his3*-*Δ5*′*] leu2*-*Δ1 rad5::KanMX*,	PR-28	Leu^+^ transformant of YB441
YB444	*MATα* *ura3-52 his3*-*Δ200 ade2-101 trp1-Δ1 gal3 trp1::[his3-Δ3*′*::HOcs, his3*-*Δ5*′*] leu2*-*Δ1 rad5::KanMX*,	PR-19	Leu^+^ transformant of YB441
YB445	*MAT**a**-inc ura3-52 his3*-*Δ200 ade2-101 trp1-Δ1 gal3 trp1::[his3-Δ3*′*::HOcs, his3*-*Δ5*′*] leu2*-*Δ1 rad5::KanMX*,	PGHOT *GAL3*	Meiotic segregant of YB441 × YB163, Trp^+^ transformant
YB446	*MAT***a** *ura3-52 his3*-*Δ200 ade2-101 trp1-Δ1 gal3 trp1::[his3-Δ3*′*::HOcs, his3*-*Δ5*′*] leu2*-*Δ1 sgs1::KanMX*		Meiotic segregant from YB445 × YA286
YB447	*MAT***a** *ura3-52 his3*-*Δ200 ade2-101 trp1-Δ1 gal3 trp1::[his3-Δ3*′*::HOcs, his3*-*Δ5*′*] leu2*-*Δ1 srs2::KanMX*		Meiotic segregant from YB204 × YA287
YB448	*MATα* *ura3-52 his3*-*Δ200 ade2-101 trp1-Δ1 gal3 trp1::[his3-Δ3*′*::HOcs, his3*-*Δ5*′*] leu2*-*Δ1 srs2::KanMX, rad5::KanMX*		Meiotic segregant from YB441 and YB447
YB449	*MATα* *ura3-52 his3*-*Δ200 ade2-101 trp1-Δ1 gal3 trp1::[his3-Δ3*′*::HOcs, his3*-*Δ5*′*] leu2*-*Δ1 ubc13:::KanMX*		Meiotic segregant from YB204 × YA284
YB550	*MATα* *ura3-52 his3*-*Δ200 ade2-101 trp1-Δ1 gal3 trp1::[his3-Δ3*′*::HOcs, his3*-*Δ5*′*] leu2*-*Δ1 mms2:::KanMX*		Meiotic segregant from YB204 × YA285
YB551	*MATα* *ura3-52 his3*-*Δ200 ade2-101 trp1-Δ1 gal3 trp1::[his3-Δ3*′*::HOcs, his3*-*Δ5*′*] leu2*-*Δ1 rad5:::KanMX, sgs1::KanMX*		Meiotic segregant from YB441 × Y446
YB348	*MAT***a***/MATα ura3-52/− his3-200/− ade2-n/ade2-a trp1-1/− gal3^−^ leu2-3, 112/− GAL1::his3-5*′ *trp1::his3-3*′*::HOcs-*		This laboratory
YB554	*MAT***a***/MATα ura3-52/− his3-200/− ade2-n/ade2-a trp1-1/− gal3^−^ leu2-3, 112/− GAL1::his3-5*′ *trp1::his3-3*′*::HOcs rad5::KanMX/−*		This laboratory

At least three independent experiments were done for each DNA-damaging agent. We reported the spontaneous recombination frequencies [number of His^+^ recombinants per colony forming unit (CFU)] and recombination frequencies obtained after exposure to DNA-damaging agents (stimulated frequency). The average net frequency of His^+^ recombinants was determined by first subtracting the spontaneous frequency from the stimulated frequency f or each experiment and then taking the average. Statistical significance was determined by the Student's t-test [39].

### 2.5. Induction of HO endonuclease

pGHOT-*GAL3* [5], containing the HO gene under *GAL* control, was introduced into wild type and *rad5* mutant strains by selecting for Trp^+^ transformants. After growth in SC-TRP medium, cells were diluted 1:10 in YP Lactate and incubated for a minimum of 12 h. At a density of 10^7^ cells/mL, glucose or galactose was added to a final concentration of 2%, to either repress or induce the expression of HO endonuclease, respectively. After 2 h, cells were plated directly on YPD medium for viability and on SC-HIS to measure recombination. Colonies appearing on YPD medium were replica plated on SC-TRP to measure the number of Trp^+^ colonies containing the pGHOT-*GAL3* plasmid.

## 3. Results

### 3.1. Spontaneous rates of unequal sister chromatid recombination do not change in mutants defective in posttranslational PCNA modifications but are increased in sgs1 and srs2 mutants, compared to wild type

Genes involved in the post-translational modification of PCNA include, *RAD5*, *UBC13*, and *MMS2*, while genes involved in the resolution of recombinational intermediates include *SGS1*. We tested the model whether DNA damage-associated recombination between sister chromatids essentially occurred by a template switch mechanism followed by resolution by *SGS1*, as suggested in Putnam et al. [40]. We first determined whether the rates of spontaneous unequal SCE were affected by deleting *RAD5*, *SRS2*, *SGS1*, *UBC13*, and *MMS2* (Table 2). The rate of spontaneous SCE observed in wild-type was similar to rates observed in previous publications. The rates of spontaneous SCE in *rad5*, *ubc13*, and *mms2* mutants were similar to wild type (Table 2), suggesting that another mechanism, besides template switching, was required for spontaneous recombination events. Rates of spontaneous recombination in the *srs2* and the *sgs1* mutants were elevated two-fold and six-fold, respectively, consistent with results that *SRS2* represses recombination between direct repeats [41]–[43] and that *SGS1* resolves recombination intermediates so that cross-overs do not occur [28],[43]. Interestingly, *sgs1 rad5* double mutant still exhibited a hyper-recombination phenotype (Table 2), suggesting that some recombination events observed in the *sgs1* mutant did not proceed by a template-switch mechanism. These results indicate that rates of spontaneous uSCE are not affected by mutations in genes that function in PCNA ubiquitination.

**Table 2.** Rates of spontaneous, mitotic recombination in *rad5*, *sgs1*, *srs2*, *mms2*, *ubc13* mutants.

Genotype (Strain)	Rate^c^	Ratio^d^
**Strains to measure uSCE ^a^**	×10^−6^	
Wild type (YB163)	0.81 ± 0.06	1
*rad5* (YB441)	1.4 ± 0.08	1.8
*sgs1* (YB446)	4.7 ± 0.7	**5.9**
*srs2* (YB447)	2.0 ± 0.03	2.5
*ubc13* (YB449)	0.73 ± 0.2	0.9
*mms2* (YB550)	0.95 ± 0.05	1.2
*rad5 sgs1* (YB551)	3.5 ± 0.04	**4.4**
**Strains to measure translocations ^b^**	×10^−8^	
Wild Type (YB348)	3.0 ± 0.8	1
*rad5* (YB554)	10.5 ± 8.1	3

^a^ All strains contain *his3-5′ his3-3′::HOcs* and are isogenic to S288C, see Table 1 for full genotype.

^b^ All strains contain *trp1*:: *his3-3′::HOcs* and *GAL1*:: *his3-5′*, see Table 1 for complete genotype.

^c^ Number of recombinants per cell division, N ≥ 2.

^d^ Rate in mutant/wild type. Bold print indicates significant difference compared to wild type (P < 0.05).

### 3.2. DNA damage-associated uSCE events are decreased in rad5 mutants after exposure to UV, the UV-mimetic agent 4-NQO, and the X-ray mimetic agent MMS

**Figure 2.**
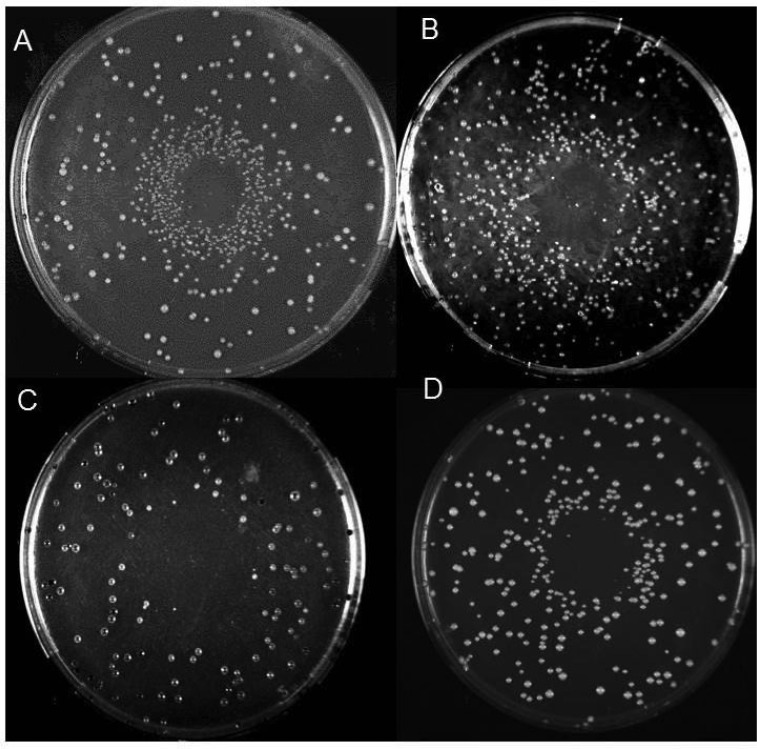
Plate assay demonstrating the DNA damage inducibility of SCE in the wild type strain but not in *rad5* strains. Each SC-HIS plate contains a lawn of 10e7 cells, and the chemical agent 4-NQO (0.5 µL 14 mM) was spotted in the center. The plates were incubated at 30^o^C for three days. (A) Wild type (YB163), (B) *sgs1* (YB446), (C) *rad5* (YB441), (D) *ubc13* (YB449). Complete genotypes are given in Table 1.

Stimulation of unequal SCE events after exposure to chemical DNA damaging agents can be observed using a halo assay, in which cells are exposed to a DNA damaging agent that diffuses from the center of the plate (Figure 2). As in previous studies [8], we observed a dense halo of chemically-induced His^+^ recombinants after wild-type cells were plated on SC-HIS and exposed to either MMS, a X-ray mimetic agent, or 4NQO, a UV-mimetic agent. However, we did not observe a strong ring of stimulated colonies in the *rad5* mutant after cells were exposed to either MMS or 4-NQO. A similar result was observed in a *rad5* diploid mutant to monitor uSCE (data not shown). These data indicate that DNA damage-associated SCE resulting from continual exposure to chemical agents is dependent on *RAD5*.

**Figure 3.**
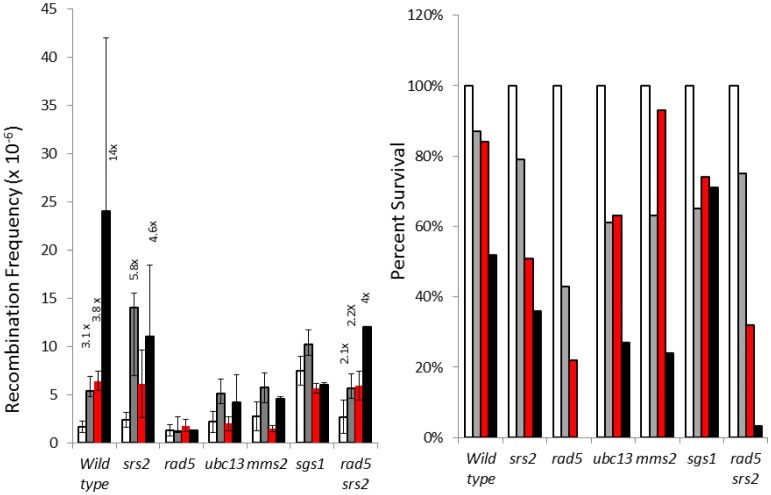
uSCE frequencies in wild-type, *srs2, rad5, ubc13, mms2, sgs1, rad5 srs2* strains after exposure to either MMS or 4-NQO. The recombination frequencies are shown in the left figure and the percent survival is shown in the right figure. Average spontaneous frequencies are represented by white bars, MMS (0.02%)-associated frequencies are under the gray bar, NQO (1 M)-associated frequencies are under the red bar, and NQO (10 M)-associated frequencies are under the red bar; N > 3. Complete genotypes of wild-type (YB163), *srs2* (YB447), *rad5* (YB441), *ubc13* (YB449), *mms2* YB450), *sgs1* (YB446), *rad5 srs2* (YB448) strains are given in Table 1. The percent survival is calculated as (Total CFU after exposure/Total CFU before exposure) × 100%. Significant increases are shown above the bars (DNA damage-associated frequency/spontaneous frequency), P < 0.05.

**Figure 4.**
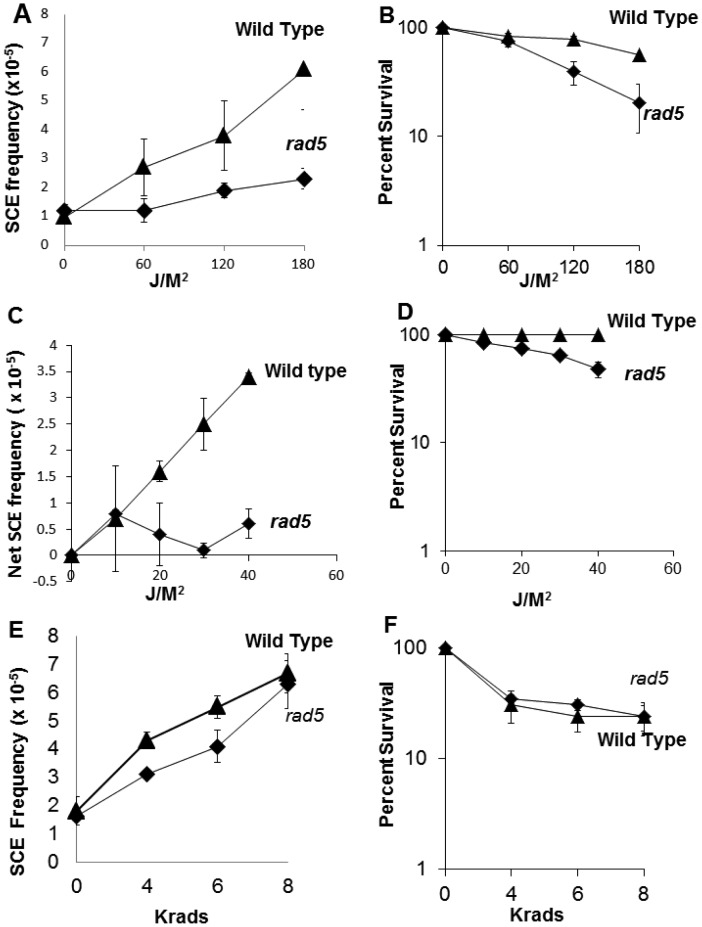
SCE frequencies in wild-type and *rad5* strains after exposure to UV and X-ray radiation. The panels on the left (A, E) plot the radiation-associated SCE frequencies against either the UV (A) or the X-ray (E) radiation dose. The net UV-associated SCE frequency was plotted against the UV dose in panel (C). The panels on the right (B, D, F) plot the percent survival against the dose. Triangles represent data points for wild type and diamond represent data points for the *rad5* mutant.

To quantify DNA damage-associated recombination in *rad5* mutants after acute exposures to chemical agents, we measured frequencies of DNA damage-associated frequencies of SCE in *rad5* mutants. In wild type cells, we observed a 3–4 fold increase of SCE after chemical exposures that yield <50% toxicity, while 14-fold increases were observed at higher exposure levels (Figure 3), consistent with previous studies [8],[11]. However, frequencies of unequal SCE in *rad5* mutants did not increase after exposure to either 4-NQO or MMS. Exposure to 10 M 4NQO conferred greater than 95% lethality, rendering it difficult to accurately assess DNA damage-associated recombination. These data indicate that *rad5* mutants are defective in DNA damage associated SCE after exposure to DNA damaging agents that produce DNA bulky adducts. Considering that many potent chemical recombinagens, such as 4-NQO, are UV radiation mimetic agents, we next (Figure4) determined whether UV exposure stimulated recombination in *rad5* mutants. We observed a 3–4 fold increase in recombination in wild type but not in *rad5* cells after exposure to 120 J/M^2^ of UV radiation. However, since the *rad5* mutant is UV sensitive, we also exposed *rad5* cells to lower doses of UV, after which the survival percentages were approximately 50%. Our data indicates that even at exposure to low UV doses, DNA damage-associated SCE requires *RAD5* (Figure 4).

### 3.3. Rad5 mutants are not defective in X-ray or HO-endonuclease associated uSCE events

Because some MMS-associated DNA damage is radiomimetic, we asked whether uSCE recombination was also stimulated by X rays in the *rad5* mutant. After exposure to 4, 6, and 8 krads of X-rays we observed that X-ray-associated recombination occurred in both wild-type and *rad5* mutants; the maximum increase was three-fold, compared to the frequency obtained after no exposure (Figure 4). Since X-rays also induce single-strand nicks in DNA, we directly generated a DSB at the site of the recombination substrate using a galactose-inducible HO endonuclease. Whereas we observed a 13-fold increase in wild type, we observed a 36-fold increase in the *rad5* mutants; the viability after HO induction was the same (Table 3). Although the increase in HO endonuclease-induced SCE is not understood, previous reports have suggested that homologous recombination is enhanced in *rad5* mutants if non-homologous recombination is not efficient [44]. Thus, both X rays and HO-endonuclease generated DSBs can stimulate SCE in both *rad5* and wild type strains, indicating that DSB-associated uSCE recombination is *RAD5*-independent.

### 3.4. Both rad5 helicase function and ubiquitin ligase functions are required for 4NQO-associated SCE

The Rad5 protein has multiple domains; it functions both as a helicase and an ubiquitin ligase [34]. We therefore asked whether a *rad5* mutant that retained the helicase function or ubiquitin ligase function was still proficient at DNA damage-associated SCE. Pages et al. [34] had previous constructed a *rad5* variant containing the D681, E682/AA mutations conferring a deficiency in the helicase function, and a *rad5* variant containing C914, C917/AA in the C3HC4 ring-finger motif conferring a deficiency in the ubiquitin ligase function; the variants are present on plasmids pR-30 and pR-19, respectively, while *RAD5* is present on pR-28. We introduced these three plasmids into *rad5* mutants and measured DNA damage-associated recombination after exposure to 10 M 4NQO (Table 4). While we observed a nine-fold increase in 4NQO-associated recombination in either a *rad5* strain containing pR28 (*RAD5*) or in a wild type strain, we only observed a four-fold and three-fold increase in 4NQO-associated SCE in *rad5* deletion mutants containing pR-19 (*rad5* C914, C917/AA) or pR-30 (*rad5* D681, E682/AA), respectively. Although this would suggest that both ubiquitin ligase and helicase functions are required for 4NQO-associated recombination, Choi et al. [45] suggest that the Rad5 helicase motif important for ATP binding is also required for PCNA polyubiquitination.

**Table 3.** Stimulation of SCE by HO-induced DSBs in wild type and *rad5* haploid strains.

Genotype*^a^* (Strain)	% Viability after HO induction*^b^*	His^+^ recombinants/Trp^+^CFU × 10^5^		Fold increase*^e^*	Ratio^f^
		Before HO	After HO		
		induction*^c^*	induction*^d^*		
Wild type (YB163)	84 ± 18	5.8 ± 1.0	76 ± 13	13	1
*rad5* (YB445)	76 ± 26	8.1 ± 2.6	304 ± 13	36	3.3

^a^ For complete genotype, see Table 1.

^b^ Trp^+^ CFU after HO induction/Trp^+^ CFU before HO induction × 100%.

^c^ His^+^ recombinants before HO induction/Trp^+^ CFU before HO induction.

^d^ His^+^ recombinants after HO induction/Trp^+^CFU after HO induction.

^e^ His^+^ frequency after HO induction/His^+^ frequency before HO induction.

^f^ Fold increase in mutant/Fold increase in wild type.

**Figure 5.**
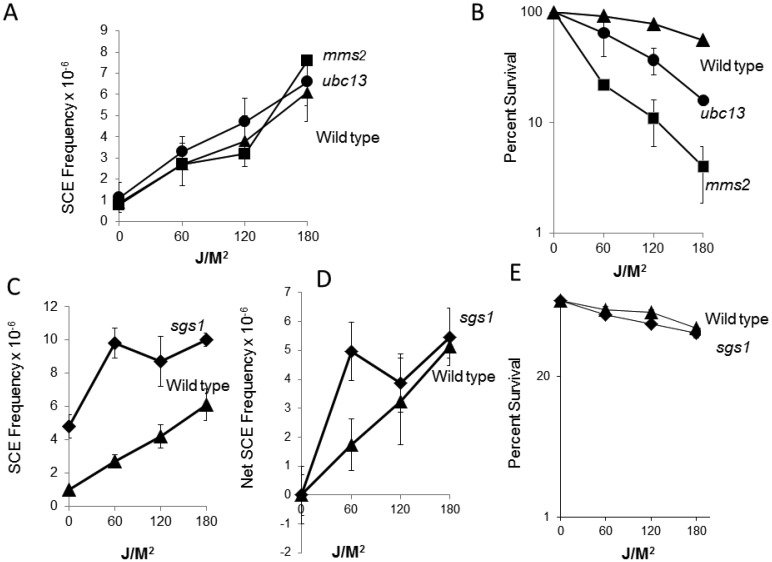
SCE frequencies in *ubc13*, *mms2* and *sgs1* mutants after exposure to UV. The panels on the left (A, C, D) plot the radiation-associated frequencies against the radiation dose. The panels on the right (B, E) plot the survival percentages against the dose. Triangles represent data points for wild type, diamond represent data points for the *sgs1* mutant, solid circle represents *ubc13* mutant, and square represents *mms2* mutant. Symbols may obscure the error bars.

If PCNA modification is important in DNA damage-associated SCE, then either *UBC13* or *MMS2*, which are also required for PCNA polyubiquitination and error-free DNA damage tolerance, should also be defective in DNA damage-associated SCE. Our data indicate that both *ubc13* and *mms2* mutants are defective for MMS and 4NQO-associated SCE recombination; however, unlike *rad5* mutants, we still observed some DNA damage-associated SCE in the halo assay (Figure 2). Interestingly, we observed similar frequencies of UV-associated SCE recombination in *ubc13*, *mms2*, and wild type (Figure 5). These data indicate that PCNA polyubiquitination is still important for DNA damage-associated SCE after cells are exposed to either MMS or 4-NQO; however neither *UBC13* nor *MMS2* are required for UV-associated SCE recombination. Thus, the genetic requirements for UV-associated SCE and for MMS and 4NQO-associated SCE recombination are different.

### 3.5. Sgs1 and srs2 deletions have opposing effects on rad5-associated uSCE

*SGS1* has been suggested to function downstream of *RAD5*, converting Holliday intermediates into non-recombinant products. We would thus predict that more DNA damage-associated SCE events would occur in *sgs1* mutants, compared to wild type. We observed that frequencies of UV-associated SCE peaked after 60 J/M^2^ exposures in the *sgs1* mutant, while the survival was only slightly reduced, compared to wild type (Figure 5). However, we observed that *SGS1* was required for both MMS and 4NQO-associated recombination (Figure 3). The data are consistent with a previous study that MMS-associated SCE recombination is *SGS1*-dependent [46] and suggest that *SGS1* may also function early in DNA damage-associated recombination between sister chromatids.

*SRS2* functions to channel potential recombinogenic lesions into DNA damage tolerance pathways that do not involve recombination [47]. We observed that *srs2* deletion could also suppress the *rad5* deficiency in DNA damage-associated SCE after exposure to low concentrations of MMS and 4NQO, and the recombination frequencies observed after DNA damage exposure for both wild type and the *rad5 srs2* double mutant were similar (P > 0.05). However, the DNA damage sensitivities of the *rad5 srs2* mutant were only partially suppressed (Figure 3), indicating that *SRS2* may also promote recombination [48]. These results indicate that *RAD5* is not required for DNA damage-associated SCE after exposures to low concentrations of the DNA damaging agent in *srs2* mutants.

**Table 4.** Requirement of ubiquitin-ligase and ATpase-associate Rad5 functions for DNA damage-associated SCE.

Strain and plasmid, (*RAD5* allele and alias)^a^	Survival^b^	Spont Freq (×10^−5^)^c^	4NQO-associated Freq (×10^−5^)^d^	Fold Induction^e^
*RAD5* (YB163)	47%	2.7 ± 0.9	24 ± 18	9
*rad5* + pR19 (*rad5*-C914, C917/AA, YB444)	43%	1.5 ± 0.5	6 ± 2.6	4
*rad5* + pR28 (*RAD5*, YB443)	39%	1.7 ± 0.4	19 ± 13	9
*rad5* + pR30 (*rad5* D681, E682/AA, YB442)	34%	1.5 ± 0.7	4.1 ± 2	2.7
*rad5* (YB441)	<0.3%	1.3 ± 0.6	1.3	1

^a^ For complete genotype, see Table 1.

^b^ Survival = Total CFU after exposure/Total CFU before exposure ×100%.

^c^ His^+^ recombinants/Total CFU (×10^−5^), N ≥ 3.

^d^ His^+^ recombinants/Total CFU (×10^−5^), N ≥ 3.

^e^ SCE frequency after exposure/SCE frequency before exposure.

### 3.6. DNA damage-associated homologous recombination is enhanced between ectopic sequences

**Figure 6.**
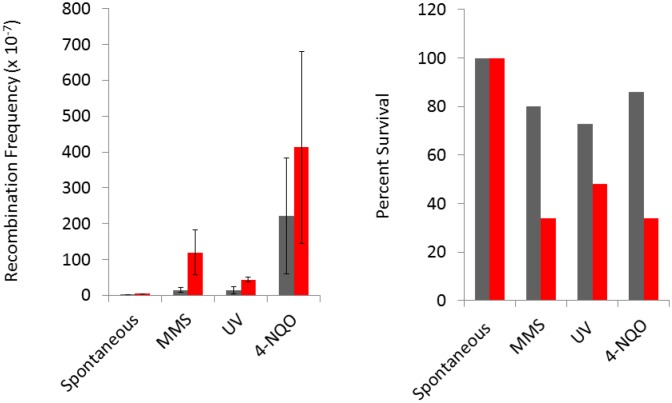
Translocation frequencies in wild-type and *rad5* diploid strains after exposure to either MMS (0.02%), UV (30 J/M^2^) or 4-NQO (10 M). The recombination frequencies are shown in the left and the percent survival is shown in the right panel. Black bars represent recombination frequencies and survival percentages obtained from the wild type strain (YB348) and red bars represent those obtained from the *rad5* strains (YB554); N > 3. Complete genotypes of wild-type (YB348), *rad5* (YB554) strains are given in Table 1. The percent survival is calculated as (Total CFU after exposure/Total CFU before exposure) × 100%. Spontaneous frequencies were (1.3 ± 0.7) × 10^−7^ for the wild type and (4.3 ± 0.7) × 10^−7^ for the *rad5* diploid.

One explanation for the *RAD5-*dependence for DNA damage-associated recombination between *his3* fragments is that *RAD5* is required for DNA damage-associated recombination between the *his3* fragments, regardless of their position in the genome. To test this explanation, we measured both spontaneous and DNA damage-associated recombination in a strain where the *his3* recombination substrates were positioned on non-homologous chromosomes (Figure 1). The rate of spontaneous recombination that generates translocations was not different in the *rad5* strain, compared to wild-type (Table 2). We then measured DNA damage-associated recombination between *his3* recombination substrates on non-homologous chromosomes after exposure to MMS (0.02%), 4-NQO (10 M), and UV (30 J/M^2^). Compared to wild type strains, we observed a three-fold difference in UV-associated translocation frequencies in *rad5* diploids after 30 J/M^2^ exposure (Figure 6) and a significant increase (P < 0.05) in the translocation frequencies in the *rad5* diploid after exposure to MMS, compared to the MMS-associated translocation frequencies in wild type. These data indicate that *RAD5* requirement for recombination between *his3* recombination substrates depends on their position in the genome.

## 4. Conclusion

Exposure to potent recombinagens, such as MMS, 4NQO, and UV, can increase frequencies of homologous recombination events occurring between sister chromatids, homologs, and repeated sequences on non-homologous chromosomes (ectopic recombination). However, it is unclear whether the major mechanism by which many bulky DNA adducts stimulate recombination at different sites in the genome is the same. For example, DNA-damage-associated SCE could occur by template switching, recombination initiated by collapsed DNA replication forks, or DSB-initiated gap repair. Multiple studies support the idea that *RAD5*-mediated template switch mechanisms are involved in the generation of recombination intermediates and that such intermediates are suppressed by *SGS1* [18],[25]. *RAD5*-associated functions include PCNA polyubiquitination and ATPase activity [34]. Here, we measured frequencies of spontaneous and DNA damage-associated uSCE in wild type and template switch mutants of budding yeast after exposure to X-rays, HO-induced DSBs, UV, MMS, and 4-NQO. We derived three major conclusions from our results. First, *RAD5* is required for UV, MMS, or 4-NQO-associated uSCE but is not required for uSCE stimulated by ionizing radiation or HO endonuclease-induced DSBs. Second, MMS and 4NQO-associated uSCE but not UV-associated SCE required *SGS1*, *UBC13*, or *MMS2*, suggesting that genetic requirements for template switching depend on the type of DNA adduct. Third, *RAD5* is not required for spontaneous SCE nor for the hyper-recombination between SCE observed in *sgs1* mutants. This is the first study to show that template switch mechanisms are involved in suppressing DNA damage-associated homologous recombination between repeated sequences present on non-homologous chromosomes.

These conclusions were based on assays using tandem *his3* recombinational substrates or *his3* fragments located on non-homologous chromosomes. The results may appear to contradict observations derived from recombination assays using non-tandem or inverted repeats, or plasmid-based recombination assays [40],[48],[49]. However, our previous studies have indicated that the *RAD* gene and checkpoint requirements for spontaneous or DNA damage-associated recombination differ depending on both the arrangement and the location of the *his3* fragments [4]. Thus, further studies are necessary to determine whether similar results would be obtained using alternative assays to monitor sister chromatid recombination.

The multiple *RAD5*-dependent pathways involved in DNA damage-associated SCE correlate with observations that recombinogenic agents that generate DNA replication blocks have different effects on replication fork progression *in vivo*. For example, UV slows DNA replication progression *in vivo* but does not stall replication at localized replication origins, while 4NQO-associated DNA damage stalls DNA replication at localized DNA replication origins [18]. We observed that *UBC13* and *MMS2* are required for 4NQO-associated SCE, while these genes are not required for UV-associated SCE. The mechanism by which UV stimulates recombination between sister chromatids involves cohesins [15],[50] but little is known how 4NQO stimulates recombination. Thus, the correlation between the nature of the DNA block and the requirement for *UBC13* and *MMS2* merits further investigation.

A previous model for template switching suggests that *SGS1* functions downstream of *RAD5* in resolving recombination intermediates so that cross-overs are avoided [40]. If recombination between tandem *his3* fragments also followed this pathway, then we would also expect to see higher frequencies of DNA damage-associated SCE events in *sgs1* mutants, compared to wild type. Interestingly, we only observed higher DNA damage-associated frequencies after exposure to 60 J/M^2^ UV dose; at higher UV doses we observed similar DNA damage-associated frequencies of recombination. Both 4NQO and MMS-associated uSCE were *SGS1* dependent, consistent with similar experiments [46]. Eukaryotic RecQ helicases, analogous to Sgs1, have been previously suggested to reverse collapsed DNA forks [51]; however, these helicases may generate single-stranded and not double-stranded DNAs from collapsed forks in the presence of single-strand binding proteins [52]. Alternatively, *SGS1* is required for Rad53 activation [53],[54]; at replication blocks, *RAD53* is required for chemical damage-associated [55], but not UV-associated uSCE [6], and lower levels of Rad53 activation in *mec1* hypomorphs does not reduce UV-associated uSCE [56]. One possibility is that activated Rad53 phosphorylates Rad55, enabling timely progression of S phase in the presence of DNA-blocking lesions [57], while UV-associated recombination may involve nucleases, such as Mus81, that cleave stalled replication forks [58],[59]. These studies suggest a correlation between the *SGS1* and *RAD53* requirements for DNA damage-associated recombination.

Our observation that spontaneous and *sgs1*-associated spontaneous hyper-recombination between tandem *his3* fragments is *RAD5*-independent suggest that alternative mechanisms, besides template switching, are involved in recombination between tandem *his3* fragments. These alternative mechanisms may include replication slippage and break-induced replication [43]. Interestingly, while DNA damage-associated uSCE is abolished in *rad51* mutants, *sgs1*-associated hyper-recombination between *his3* fragments is only partially suppressed by *rad51* mutation [43]. These results are in agreement with observations that replication intermediates may accumulate by a *RAD51*-independent mechanisms [60]. The observations underscore differences between spontaneous and DNA damage-associated recombination pathways, as previously observed [8]. Thus, *SGS1* may abort sister chromatid recombination intermediates that are generated by multiple mechanisms.

The *RAD5* function to promote template switching also suppresses DNA damage-associated homologous recombination between sequences located at ectopic sites. This result is consistent with observations that higher levels of gross chromosomal rearrangements are generated in *rad5* mutants, compared to wild type [61],[62]. Although the consequences of failed template switching have not been fully explored, one possibility is that more DSBs are generated when template switching fails [61],[62]. In support of this notion, exposure to DNA damaging agents stimulate more homology-directed translocations in the *rad5* diploid, compared to wild type (Figure 6). Interestingly, ionizing radiation but not 4NQO stimulates more homology-directed translocations in the *rad9* mutant defective in G2 checkpoint control, compared to wild type [9]. These results suggest that DSBs are an indirect consequence of 4NQO-associated DNA damage and are generated at a higher frequency in *rad5* strains.

In conclusion, we have shown that the major pathway by which MMS, 4NQO, and UV stimulate uSCE is *RAD5*-dependent, while DSB-initiated uSCE occurs by *RAD5*-independent mechanisms. MMS and 4NQO-associated but not UV-associated uSCE are *UBC13*, *MMS2*, and *SGS1*-dependent, suggesting that there are multiple *RAD5*-dependent mechanisms involved in DNA damage-associated SCE. Considering that template switch mechanisms are important in tolerating low levels of DNA damage [63], it will be important to determine whether the human [64],[65] and plant [66] homologs of *RAD5* share similar functions.
